# Discomfort and unease of the subject in the interpretation movement of a Tuberculosis questionnaire

**DOI:** 10.1590/0104-1169.3522.2507

**Published:** 2014

**Authors:** Rarianne Carvalho Peruhype, Laís Mara Caetano da Silva, Elisângela Gisele de Assis, Ana Carolina Scarpel Moncaio, Lenilde Duarte de Sá, Pedro Fredemir Palha

**Affiliations:** 1 Doctoral students, Escola de Enfermagem de Ribeirão Preto, Universidade de São Paulo, WHO Collaborating Centre for Nursing Research Development, Ribeirão Preto, SP, Brazil; 2 PhD, Associate Professor, Universidade Federal da Paraíba, Paraíba, PB, Brazil; 3 PhD, Associate Professor, Escola de Enfermagem de Ribeirão Preto, Universidade de São Paulo, WHO Collaborating Centre for Nursing Research Development, Ribeirão Preto, SP, Brazil

**Keywords:** Semantics, Tuberculosis, Health Personnel

## Abstract

**OBJECTIVE:**

to propose a discussion about traces of the derivation of meanings, the subjects' discomfort and resistance when they are called upon to signify a questionnaire on the transfer of the Directly Observed Treatment of Tuberculosis policy, in order to reveal the limitations of closed questionnaires in the subject's interpretation process.

**METHOD:**

health professionals from a Primary Health Care Unit in Porto Alegre/RS were interviewed and some excerpts from the interviews were investigated in the light of French Discourse Analysis.

**RESULTS:**

resistance, discomfort, slips, silencing and the derivation of meanings were observed in the subjects' interpretation.

**CONCLUSION:**

the interpretation process has multiple meanings and varies from subject to subject. The questionnaire, as a prototype of the logically stabilized universe, fails when the purpose is to control the interpretation. Its isolated use in health research can entail inexactness or incompleteness of the collected data. Therefore, its use associated with qualitative research techniques is ideal.

## Introduction

A proposed analysis of the contradiction, rupture, derivation of meanings in discourse is, above all, a proposed analysis of the subject, of the mistake, of the unease, of the discomfort. We are referring to the subject addressed by Michel Pêcheux, which entails the need to dominate the discursive knowledge, to (un)identify oneself, to resist, to (re)signify oneself, to make oneself understood. This subject signified by the memory (referring to the social instead of the cognitive memory), affected by the ideology, by the history and its facts that claim meanings, by the processes and production conditions of language, as well as by the language, not understood as an abstract system, but as means for humans in the world to signify and produce meanings^(^ 1 ^)^.

It is known that subjects signify and (re)signify a fact in multiple and diversified ways, and that made us question how health professionals who work directly in the prevention and control of tuberculosis (TB) would signify a questionnaire with questions about the Directly Observed Treatment (DOT) of tuberculosis. In this research activity, some basic concepts of French Discourse Analysis (DA) were mobilized, mainly according to Pêcheux, which are considered fundamental for the development of this research.

Talking about DA means thinking of a space of correlation among three areas: "Linguistics", "Psychoanalysis" and "Historical and Dialectical Marxism". Discourse is considered as "the place where one can observe the relation between language and ideology, understanding how the language produces meanings by/for the subjects"^(^ 1 ^)^. According to Discourse analysis, language is opaque, i.e. not transparent, and the subject is affected by the historicity, ideology, the symbolic, the memory, the unconscious. In its signification process, there are countless possibilities of (re)signification, which is an important characteristic to interpret and understanding the object analyzed in this research.

As regards the DA concepts, initially, we consider the logically stabilized universe, which the questionnaire fits into as a prototype, as well as the formalist-logical trend and the distinction between the semantic concept for this area of Linguistics and for Discourse Analysis.

The logically stabilized universe is understood as the universe in which "interpretation is prohibited, implying the regulated use of logical propositions (True or False)"^(^ 2 ^)^; "it is supposed that any speaking subject knows what (s)he is talking about"^(^ 2 ^)^ and is characterized by logical homogeneity and univocal simplification. That is the subject's universe of standardization, of strictness, called the "pragmatic subject": "The pragmatic subject - that is, each of us, the 'private simple' in view of the different urgencies of their life - has an imperative need for logical homogeneity': that is marked by the existence of these multiple and small portable logical systems that range from the daily management of existence (examples in our civilization are writing sets, keys, agenda, papers etc.) to the "big decisions" of social and affective life (I decide to do this and not that, to answer X and not Y etc...)" ^(^ 2 ^)^.

This logical reductionism arouses a feeling/illusion of dominion in the subject, of complete control of the meaning, discourse, saying, understanding, being. That is the subject's representation of practical objectivity, who needs a linear, "semantically normal world"^(^ 2 ^)^, without derivations of meaning or resistances that cannot be controlled.

Escaping from this characteristic of the logically stabilized universe, in DA, semantics is aimed at addressing meaning with its possible slips, dislocations and derivations, defined as that meaning whose "expression, proposition does not exist by itself, and can only be constituted in relation to the production conditions* of a given statement, as it changes according to the ideological formation** of who (re)produces and of who interprets them. The meaning is never given, it does not exist as an end product, a result of a possible language transparency, but is always in course, moving and is produced in a historical-social determination, entailing the need to discuss effects of meaning"^(^ 3 ^)^.

That is the discursive semantics of interest, which evokes meaning as a field of multiple and countless possibilities of (re)significations that are not preset by language and go beyond the semantics Linguistics emphasizes. According to Pêcheux, "semantics does not conceive language as a representation of the world, and therefore admits neither the transparency of language nor the subject's exteriority in relation to the language"^(^ 4 ^)^. The semantics of formalist-logical linguistics, on the other hand, only values clarity, grammatical organization and form. This trend emphasizes pure forms, highlights laws and underlines the central role of syntax in grammar and these elements' role in the interpretative semantics^(^ 5 ^)^.

In view of the concepts of semantics and meanings presented, we return to the research questionnaire, considered as a prototype of the logically stabilized universe, because it contains characteristics of objectivity, logical homogenization, generalization, supposing that the subject who is to be interviewed knows what (s)he is talking about and because it creates the illusion of controlling the meanings. Nevertheless, "the problem raised is that these discursive spaces do not foresee any sliding meanings, that the subjects resist the enforcements of their laws, that the real is the object of multiple interpretations..."^(^ 6 ^)^. Hence, although one cannot deny the importance of using questionnaires, whether validated or not, in the empirical sciences, it is equally important to highlight that this apparent control of the meanings pictures an illusory situation of mastery which the subject's own action needs to undo in his/her interpretation movements.

Thus, the objective in this article is to analyze, based on the theoretical premises of Pêcheux's Discourse Analysis, how the health professionals signified this research instrument.

## Method

A qualitative study was developed through the analysis of discourse collected through audio-recorded interviews. It should be highlighted that this study did not aim to discuss the results obtained based on the application of the questionnaire, but the health professionals' perception of the research instrument and how they felt when they answered it.

It is highlighted that this instrument is part of the multicenter project entitled "Assessment of the Transfer of Directly Observed Treatment Health Policies in some cities in the South, Southeast, Northeast and North", approved by the Research Ethics Committee at the University of São Paulo at Ribeirão Preto College of Nursing (EERP) (CAAE 01197312.3.0000.5393), in compliance with the requirements of the Helsinki Declaration. Authorization for data collection was obtained in each of the research scenarios, that is, Porto Alegre/RS, Ribeirão Preto/SP, João Pessoa/PB and Manaus/AM, respectively, and the interviews were held after the research subjects had given their authorization and signed the Informed Consent Form, which guaranteed the secrecy and anonymity in accordance with National Health Council Resolution 466/12.

Both the application of the questionnaire and the interviews with the health professionals were part of the semantic validation phase of this instrument, involving 24 health professionals (physicians, nurses, nursing technicians and auxiliaries), six per research site.

It is important to consider that this study will be restricted to the analysis of some excerpts from interviews involving six health professionals from a Primary Health Care Unit in the city of Porto Alegre. In this scenario, approval was obtained (from the Research Ethics Committees of the Municipal Health Department and the Grupo Hospitalar Conceição, co-participants in this research) for the specific sub-project in this region. Although the research corpus comprises the answers of six subjects, we will focus on excerpts from only two subjects' discourse, considering that DA does not aim for an exhaustive horizontal analysis or that considers the full extent of the research problem, considering that it is not exhausted and that a discourse is always produced in relation to others. Instead, it rests on vertical exhaustiveness, with a view to addressing the research objectives and the research theme in depth^(^ 1 ^)^.

As regards the structure, the instrument consists of 49 items, to be assessed on a modified Likert scale with the following items: I completely disagree; I disagree; I neither agree nor disagree; I agree; I completely agree. These items were grouped in three main categories ("information", "knowledge" and "innovation") and address structural, contextual, financial and human resource aspects, as well as elements that are considered important for the transfer and operation of the DOT policy at the health service. DOT can be considered as the monitoring and direct supervision of the medication intake by the TB patients from Mondays to Fridays in the Attack Phase of the disease and at least three times per week when in the Maintenance Phase. Either health professionals or any other person can do this monitoring, provided that they are properly trained and preferably work under the supervision of nurses.

Concerning the semantic validation phase, in a comprehensive validation proposal of research instrument, its objective is to make the language clearer and more accessible, as well as to avoid multiple interpretations of an assertion, causing the respondents' lack of understanding^(^ 8 ^)^. As shown earlier, semantics is a highly relevant concept in DA and is fundamental in the signification process by/for the subject of the discourse.

In the semantic validation process of the instrument, two distinct moments can be highlighted: initially, the questionnaire was applied to the health professionals and, then, forms were completed with (general and specific) impressions about the questionnaire and individual interviews were held, aiming to collect additional information on the applied instrument. Using the forms, the objective was to assess the importance and understanding of the questionnaire items according to the health professionals, as well as the need for modifications, adaptations and qualifications of the initial proposal instrument. At that moment, the research subjects answered three main questions: "Would you like to change anything in the questionnaire?"; "Would you like to add anything in the questionnaire?"; "Was there any question you did not want to answer? If yes, why?".

After applying the research instrument, completing the forms with general and specific impressions and closing off the interviews based on the three questions above, we proceeded with their transcription and analysis.

## Results and discussion

In response to question 1 ("Would you like to change anything in the questionnaire?"), one subject manifests his concern with the possibility of multiple interpretations of some items and expresses his position as follows: *I answered instinctively making my distinction (*Subject 1, high education level, nurse).

If we apply some linguistic elements and rest on the theoretical premises of Pêcheux's DA, we could say that the modal adverb *"instinctively*", which links the subject "I" to the verb "answered", makes us question what this "instinct" would be for the purpose of a discursive event and for the formulation of his statement. The phrase "*I answered instinctively"*, in our reading, can remit to the subject's activation of a memory that would make him understand the questions expressed in the questionnaire.

When he says *"making my distinction",* he could be referring to an ideological formation and to a discursive formation* and the related meanings that were mobilized, which would allow him to reach a reasoning that is considered pertinent, which would "naturally" lead him to the illusion of completeness, order and mastery of language. The possessive pronoun "*mine" *leaves traces of the discursive forgetting, explained by Pêcheux as the illusion of authorship, of original knowledge, in which the subject believes that (s)he is the primary source of a certain statement/knowledge and thinks that that is the best way to express what (s)he wants to signify^(^ 1 ^)^.

The statement "*my distinction*" also refers to the possible derivations of meanings, to the possible slips in the logically stabilized, to the possible heterogeneity hidden under fictitious homogeneity. When considering the entire sentence "*I answered instinctively making my distinction*", one could consider a certain automatism in the answer when he says *"I answered instinctively"*, creating the illusion characteristic of the logically stabilized world, in that any speaking subject knows what (s)he is talking about, through the mobilization of a personal collection over which (s)he has the alleged idea of total control. The complement "*making my distinction", *however, indicates the contradiction, the denial of this characteristics, through the polysemy, that is, the multiple meanings and the conflict of the subject who moves in this semantic network, who tries to judge for himself what is right and wrong and who asserts himself beyond the frontiers of the stabilized, making his "distinction".

The next excerpts presents remnants of the resistance and tensioning inherent in the subject. When asked whether he would like to change something in the questionnaire, he answers as follows with regard to one item about the TB patient's autonomy to decide on whether or not he wants to participate in the DOT: *I think he should not have the autonomy to decide whether he wants to or not, it would have to be imposed. He has to do the treatment. There's nothing he can do. We live in a free and democratic environment of course, but I think it's wrong here* (Subject 6, secondary level, nursing technician).

The influence of the *discursive formation health *can be observed, the imaginary formations of position and power: the health professional as the "holder" of scientific knowledge occupies a superior position in relation to the health service user, a reality that grants him the power to decide and judge on the best treatment, what should and should not be allowed, as revealed in the expression "*there's nothing he can do"*. This remits to a picture of Heteronomy, that is, "the power given, or which some professionals pretend to possess, to determine how their patients should behave, imposing their will"^(^ 9 ^)^, or to the Medicalization process and the exacerbated political intervention of medicine in society, imposing moral and behavioral standards and enhancing individuals' dependence on medical knowledge^(^ 10 ^)^.

The excerpt "*we live in a free and democratic environment of course, but I think it's wrong here*" shows the subject's resistance and (de)limitations he attributes to the words free and democratic: the patient's freedom to choose and the possibilities to express his will end when the health professional's decision starts. The conjunction "*but*", which materializes the limit, comes with the embedded literal idea of contradiction in the sentence, as well as of resistance, also emphasized through the complementary phrase "*I think it's wrong here*". The judgment of right and wrong is his, the health professional's function, as a subject in a privileged position in the knowledge hierarchy in health and escaping from this logic in the decision process is considered inappropriate. The repetition of the verb "have to" is another trait of this imposing logic, of the professional's dominion over the patient and of how the research subject established the imaginary formations of position and power.

The resistance is also expressed through the silencing, according to another excerpt from the same subject's (6) answers when he expressed his position at another moment during the interview: *Let me see... it's something like, let me see: if the TB patient does not have the autonomy to choose the modality. No, no. Have the autonomy... well, I... forget about this one... Let us see here.* (Subject 6, secondary level, nursing technician).

When saying *"well, I... forget about this one..." *the subject shows traits of unease, of resistance. The not said which says and signifies a lot, the silencing in its main characteristic of being a "significant *continuum"* ^(^ 11 ^)^, a field open to the derivation of meanings. The "*No, no*" as a literal element of contradiction, of denial and of non-acceptance precedes a sentence that reveals provocation, a factor of disequilibrium that encourages a movement of rupture, in this case the term "autonomy". The pause after the expression "*well, I..."* creates precedents to think of a complement for a voice that aims to avoid the effect of the statement, but that nevertheless is unable to escape from its polysemic linguistic nature and ends up signifying through the silence.

Many are the forms through which the subject manifests unease, discomfort, bother. The following excerpt can serve as an example: *So the middle column was kept... I don't like the middle column very much, you know? But I think we, hum, are able to take a stand, right?* (Subject 1, higher education level, nurse);

The middle column (I neither agree nor disagree) aims for an "impartiality" that bothers the subject ("*I don't like the middle column very much, you know?"*) as he signs up for what he does and any reading gestures by itself can be considered an interpretation, a judgment and a trait of partiality; "there is no observation without hypothesis, nor fact without questions"^(^ 12 ^)^.

Although the subject wants to take a stand, sometimes, the contradiction/bias leads him to different signification routes, to the (dis)construction of meanings, and even more to the impossibility of saying. The questionnaire item "I neither agree nor disagree" by itself establishes a conflict, a tension. The same is true for the subject, who takes part in this same process by saying "so the middle column was kept". In this process, he attempts to (re)signify and is confronted with the opaque, the failure of language, the discomfort, the (un)certainty. He also provides clues of his hesitation, like through the use of "*hum" *in the sentence "*But I think we, hum, are able to take a stand, right?" *for example. We notice the subject of the desire, who is anxious to master the saying and is confronted with the reality of the language, in a reality of interpretation, which "can both appease and threaten"^(^ 13 ^)^.

## Final Considerations

Although the logically stabilized universe seeks homogeneity, a single voice, logical and formalist objectivity, it fails in its proposal to control the meanings. Although the illusion of mastery is a constant in these stabilized worlds, the discursive subject moves actively in the interpretation networks, whose inner workings open up to polysemy, drift and silence.

The importance of the health sciences and other empirical sciences' use of questionnaires is undeniable, which is a research activity that can provide valuable support for the planning, organization, elaboration and qualification of programs and public policies. Nevertheless, it cannot be ignored that, no matter how complete they seem to be, the questionnaires will never fully cover the complete range of semantic aspects involved in the subject's interpretation movement and that, behind the evidence of a response, we will inevitably find the opacity of language and the subject's resistance. Therefore, we highlight the value of mixed-approach studies in health and suggest that quantitative foci, which use closed research instruments, can be complemented through qualitative analysis perspective, with a view to obtaining additional information which the use of the quantitative branch alone does not capture.

The wealth of discourse reflects in the infinite possibilities of attributing meaning, depending on where the subject is speaking, on where the analyst is speaking, on where the voices from the unconscious are speaking, on the discursive memory, on the historicity, on the ideology, on what one is speaking about, for what purpose one is speaking, what is the goal of the discourse and what is imagined through it. Therefore, one may consider that simplicity may never have taken such a complex and the obvious such as discussable and endless form.

The fact that the interlocution between health and Pêcheux's DA remains incipient turns this study into an important contribution in the expansion of scientific evidence on the theme, seeking a more in-depth analysis of the subjects discourse, in accordance with the unconscious, historicity and ideology.
